# PDGFRβ Is a Novel Marker of Stromal Activation in Oral Squamous Cell Carcinomas

**DOI:** 10.1371/journal.pone.0154645

**Published:** 2016-04-29

**Authors:** Vinay K. Kartha, Lukasz Stawski, Rong Han, Paul Haines, George Gallagher, Vikki Noonan, Maria Kukuruzinska, Stefano Monti, Maria Trojanowska

**Affiliations:** 1 Bioinformatics Program, Boston University, Boston, Massachusetts, United States of America; 2 Division of Computational Biomedicine, Boston University School of Medicine, Boston, Massachusetts, United States of America; 3 Arthritis Center, Boston University School of Medicine, Boston, Massachusetts, United States of America; 4 Division of Oral Pathology, Boston University School of Dental Medicine, Boston, Massachusetts, United States of America; 5 Department of Molecular and Cell Biology, Boston University School of Dental Medicine, Boston, Massachusetts, United States of America; INRS, CANADA

## Abstract

Carcinoma associated fibroblasts (CAFs) form the main constituents of tumor stroma and play an important role in tumor growth and invasion. The presence of CAFs is a strong predictor of poor prognosis of head and neck squamous cell carcinoma. Despite significant progress in determining the role of CAFs in tumor progression, the mechanisms contributing to their activation remain poorly characterized, in part due to fibroblast heterogeneity and the scarcity of reliable fibroblast surface markers. To search for such markers in oral squamous cell carcinoma (OSCC), we applied a novel approach that uses RNA-sequencing data derived from the cancer genome atlas (TCGA). Specifically, our strategy allowed for an unbiased identification of genes whose expression was closely associated with a set of bona fide stroma-specific transcripts, namely the interstitial collagens COL1A1, COL1A2, and COL3A1. Among the top hits were genes involved in cellular matrix remodeling and tumor invasion and migration, including platelet-derived growth factor receptor beta (PDGFRβ), which was found to be the highest-ranking receptor protein genome-wide. Similar analyses performed on ten additional TCGA cancer datasets revealed that other tumor types shared CAF markers with OSCC, including PDGFRβ, which was found to significantly correlate with the reference collagen expression in ten of the 11 cancer types tested. Subsequent immunostaining of OSCC specimens demonstrated that PDGFRβ was abundantly expressed in stromal fibroblasts of all tested cases (12/12), while it was absent in tumor cells, with greater specificity than other known markers such as alpha smooth muscle actin or podoplanin (3/11). Overall, this study identified PDGFRβ as a novel marker of stromal activation in OSCC, and further characterized a list of promising candidate CAF markers that may be relevant to other carcinomas. Our novel approach provides for a fast and accurate method to identify CAF markers without the need for large-scale immunostaining experiments.

## Introduction

It is well recognized that the tumor microenvironment, consisting of carcinoma associated fibroblasts (CAFs), endothelial cells, and immune cells, is vital for carcinoma cell proliferation, invasion and metastasis. CAFs, due to their ability to produce and dynamically modulate extracellular matrix (ECM), play a particularly important role in tumor invasion and subsequent metastatic colonization [1–4]. CAFs also produce angiogenic factors, proteases, growth factors, immune response-modulating proteins, anti-apoptotic proteins, and signaling molecules—all highly relevant to tumor biology. The cross-talk between tumor cells and CAFs is bi-directional, with fibroblasts evolving in parallel with tumor cells and undergoing phenotypic modifications in response to changes occurring in tumors [4]. The specific mechanisms underlying these complex interactions are only beginning to be elucidated and are likely to be influenced by the type of tumor and the local tissue microenvironment.

The activated tumor stroma shares some similarities with generic wound repair, as well as tissue fibrosis. It can be viewed as a biological response to a disrupted or damaged epithelial layer with stromal activation representing a repair process to restore tissue integrity and homeostasis [5]. The origin of CAFs can be diverse and involve both local and distant reservoirs. Locally, CAFs can arise from resident tissue fibroblasts, where TGFβ, as well as a stiffening matrix can promote their differentiation to alpha smooth muscle actin (αSMA)-positive myofibroblasts [2,6]. Alternative local sources may include mesenchymal or adipose-derived stem cells (MSC or ASC), as well as endothelial cells that can give rise to CAFs through endothelial to mesenchymal transition (EnMT). In some tumors, epithelial tumor cells may acquire a CAF-like phenotype through epithelial to mesenchymal transition (EMT). The contribution of bone marrow-derived MSCs and circulating CD34+ fibrocytes was also documented in several tumor models [1].

The importance of CAFs in oral cancer is supported by several reports that show correlation between the presence of αSMA-positive fibroblast cells and poor prognosis [7,8]. In a large study of OSCC patients, the abundance of myofibroblasts was the best independent predictor of patient mortality [1]. However, the source of these phenotypically-active fibroblastic cells in OSCC lesions and the mechanisms underlying their activation remain poorly understood. The progress in this field is hindered by the lack of reliable fibroblast-specific markers owing to the heterogeneity and remarkable plasticity of fibroblast cells. Furthermore, a comprehensive analysis aimed at identifying such markers using high-throughput, genome-wide expression data is yet to be performed.

Here, we report our evaluation of PDGFRβ’s role as a potential CAF marker in human OSCCs through a combination of high-throughput gene expression analyses of large primary tissue datasets and experimental validation using a panel of OSCC specimens and cell lines. To allow for an unbiased identification of fibroblast-specific markers in OSCC, we searched for genes whose expression closely associated with typical fibroblast-specific genes, namely, the interstitial collagens COL1A1, COL1A2, and COL3A1, using mRNA sequencing data derived from the cancer genome atlas (TCGA). We identified several candidate CAF-specific markers in OSCC, including receptor protein PDGFRβ, among the top hits. Subsequent analyses of OSCC specimens demonstrated that PDGFRβ was abundantly expressed in stromal fibroblasts, while it was absent in tumor cells, with greater specificity compared to other markers such as αSMA or podoplanin. By then extending the collagen-based interrogation to multiple cancer types, we highlighted the potential importance of PDGFRβ as a marker in other cancers. Overall, our data suggest that PDGFRβ can serve as a reliable marker for identification of CAFs in OSCC, and offer a list of additional candidates that may serve as an important resource for CAF detection and diagnostic strategy development in cancers.

## Materials and Methods

### TCGA RNA-sequencing data processing

RNA-sequencing (RNASeq) data was downloaded for TCGA Head and Neck Squamous Cell Carcinoma (HNSC) cancer cohort using Firehose v0.4.3 corresponding to the February 4th 2015 Firehose release. RNASeq version 2 data pertaining to Level 3 RSEM-normalized gene expression values was used. OSCC data was derived from the HNSC dataset by only including patients that corresponded to OSCC anatomic neoplasm subtypes, namely alveolar ridge, base of tongue, buccal mucosa, floor of mouth, oral cavity and oral tongue (N = 352). Adjacent morphologically normal epithelial (AE) samples were defined using TCGA sample barcoding information and used as a control group. Only genes having non-zero expression in at least one sample were retained in each of the analyzed datasets.

### Genome-wide collagen Nearest Neighbor (NN) analysis

Mean collagen expression of *COL1A1*, *COL1A2* and *COL3A1* across all samples in the OSCC dataset was computed and used as a reference for fibroblast-specific marker expression. These values were then correlated genome-wide (N = 20,243) using Pearson correlation with rigorous sample permutation-based p-value calculation to assess statistical significance for each gene (n = 1,000), and false discovery rate (FDR) used for multiple hypotheses testing correction. A ranked list of genes in decreasing order of Pearson coefficient was generated and used in subsequent analyses.

### Pan-cancer collagen NN enrichment analysis

TCGA RNASeq gene expression data pertaining to Adrenocortical carcinoma (ACC), Bladder urothelial carcinoma (BLCA), Colon adenocarcinoma (COAD), Kidney renal clear cell carcinoma (KIRC), Acute myeloid leukemia (LAML), Liver hepatocellular carcinoma (LIHC), Lung adenocarcinoma (LUAD), Lung squamous cell carcinoma (LUSC), Pancreatic adenocarcinoma (PAAD), and Prostate adenocarcinoma (PRAD) was obtained as described for the HNSC dataset (see TCGA RNA-sequencing data processing). The described NN analysis was applied to each dataset, similar to OSCC (see Genome-wide collagen Nearest Neighbor (NN) analysis), yielding a ranked list of genes for each dataset sorted in decreasing order of Pearson correlation coefficient. The top 50 genes for each cancer type were then tested for enrichment against each of the other cancer types by a Kolmogorov-Smirnoff (KS) test (using the ranked list in the given cancer type as a reference). This yielded pairwise KS D statistics for all 11 cancer types. Since enrichment for a given pair of cancer types was tested in both directions, the two values were averaged. Cancer types were then clustered using hierarchical clustering with average linkage for the agglomeration rule.

### Stromal score analysis

Correlation of stroma scores obtained using the ESTIMATE algorithm with mean collagen expression was computed (Pearson r = 0.88, p-value < 0.0001). Stroma signature scores based on the ESTIMATE [9] algorithm, pertaining to RNASeqv2 data for HNSC samples were downloaded from http://ibl.mdanderson.org/estimate/. For OSCC tumor samples that had both stroma scores and gene expression values (N = 185), the association between tumor sample stroma score and mean collagen expression was assessed by Pearson correlation.

### Tumor purity analysis

Estimates of tumor purity computed using the ABSOLUTE algorithm [10] were obtained for HNSC samples from the Broad Institute of MIT and Harvard. For the OSCC tumor samples that had both ABSOLUTE tumor purity estimates and gene expression values (N = 278), we applied a Pearson correlation to test for the association between tumor sample purity and the described mean collagen expression.

### Cell lines

Tested and authenticated CAL27 cells were purchased from ATCC. HSC-3 cells were a kind gift from Roberto Weigert (NIH, Bethesda, MD), and were described previously [11]. CAL27 and HSC-3 cells were cultured under standard conditions in DMEM (Mediatech, Herndon, VA) supplemented with 10% FBS (Sigma-Aldrich). Fibroblast culture was established from human tongue OSCC specimens (see Human oral tissue specimens). Biopsy was cut into small pieces and dissociated enzymatically by 0.25% collagenase (Worthington Biochemical Corporation, Lakewood, NJ) in DMEM with 20% fetal bovine serum. Digested tissue was placed in a culture dish in 5 ml of DMEM with 20% fetal bovine serum and grown for 3–5 days. The resulting confluent culture was subsequently passaged in DMEM with 10% fetal bovine serum.

### Real-time PCR

Total RNA was isolated using TRIzol reagent (MRC, Inc., Cincinnati, OH). Real-time PCR was performed using the StepOnePlus^™^ Real-Time PCR System (Applied Biosystems, Carlsbad, CA). Briefly, 1 μg of total RNA was reverse transcribed with random hexamers in a total volume of 20 μl using a Transcriptor First Strand cDNA Synthesis Kit (Roche, Tuscon, CA) according to the manufacturer’s protocol. The cDNA was diluted to 200 μl. The real-time PCR was carried out using SYBR^®^ Green PCR Master Mix (Applied Biosystems) with 2 μl of diluted cDNA in triplicates with glyceraldehyde 3-phosphate dehydrogenase (GAPDH) as the internal control. Real-time PCR was performed at 95°C for 10 min, followed by 40 cycles of 95°C for 15 sec and 60°C for 1 min. Melting curve analysis of PCR products confirmed the absence of secondary products. The primer sequences used for real-time PCR are available upon request.

### Human oral tissue specimens

This study has been designated exempt (IRB Protocol # H-32423) by the Boston University School of Medicine Institutional Review Board, as no human subjects were studied and experiments were performed using human oral tissue specimens. Tissue specimens were obtained from patients at Boston University Medical Center, and were acquired from scalpel-generated incisional biopsies of six tongue specimens representing benign hyperkeratosis and epithelial hyperplasia, 10 tongue ulcer specimens, as well as from surgical resections of moderately differentiated (n = 8) and poorly differentiated (n = 4) OSCCs of the lateral tongue border and of the floor of the mouth. For each condition, cytologically-normal adjacent epithelia were also obtained and analyzed. Benign epithelial hyperplasia, dysplasia, and OSCC regions, as well as adjacent epithelia, were defined by an onsite histopathologic examination and tissues were snap frozen at −80°C. A portion of the tissues were sectioned and used for hematoxylin and eosin (H&E) staining and immunofluorescence imaging. Sections (3 μm) of tissues were placed on OptiPlus Positive-Charged Barrier Slides (BioGenex), deparaffinized, treated with Retrievit-6 Target Retrieval Solution (BioGenex), and then processed. OSCC tissues were lysed for biochemical analysis.

### Immunohistochemistry and picrosirius red staining on tumor and ulcer biopsy tissues

Immunohistochemistry was performed on formalin-fixed, paraffin-embedded 8 μm oral mucosa tissue sections using the Vectastain ABC kit (Vector Laboratories, Burlingame, CA) and Vector ImmPress Rabbit or Rat-AP kit according to the manufacturer's instructions. Briefly, sections (5 μm thick) were mounted on APES (aminopropyltriethoxy silane solution)-coated slides, deparaffinized with Histo-Clear (National Diagnostics, Atlanta, GA), and rehydrated through a graded series of ethanol. Endogenous peroxidase was blocked by incubation in 3% hydrogen peroxide for 30 minutes, followed by incubation with 0.15 M glycine for 45 minutes, and blocking buffer (3% BSA) for 1 hour. Sections were incubated overnight at 4°C with antibodies against PDGFRβ (rabbit polyclonal anti-PDGFRβ, Cell Signaling, Danvers, MA), Periostin (rabbit polyclonal anti-Periostin, Abcam, Cambridge, MA) Podoplanin (rat anti-human Podoplanin, eBioscience, San Diego, CA) and Smooth Muscle Actin (biotinylated mouse monoclonal anti-SMA, NeoMarkers, Fremont, CA). For picrosirius red staining, slides were prepared similarly and were placed following rehydration in prewarmed Bouins’ solution for 1 hour at 60°C, followed by 0.1% Direct red (Sigma-Aldrich, St. Louis, MO) in saturated aqueous picric acid for 30 minutes. After brief washing in water, slides were placed in 0.1% Fast green (Fisher Scientific, Pittsburgh, PA) in water for 10 minutes, quenched in 1% acetic acid, and dehydrated through a graded ethanol series.

### Immunofluorescence staining on frozen specimen sections

For all immunofluorescence staining tumor samples were directly embedded in O.C.T. compound, flash frozen, and stored at -80°C. Staining was performed on 5 μm cryosections of tumor. Briefly, slides were blocked with a blocking solution (3% BSA [Sigma-Aldrich], and 0.1% Triton^®^ X-100 in PBS) for 2 h. After washing, tissue sections were incubated at 4°C overnight with primary antibodies: rabbit polyclonal PDGFRB (Cell Signaling, Danvers, MA), mouse anti-E-Cadherin (BD Biosciences) and goat polyclonal VE-Cadherin (Santa Cruz, CA). After washing, tissue sections were incubated with secondary Ab: Alexa fluor 488 donkey anti-rabbit (Invitrogen, Grand Island, NY), Alexa fluor 594 donkey anti-mouse, and Alexa fluor 594 donkey anti-goat (Invitrogen, Grand Island, NY) for 1.5 h. Coverslips were mounted on slides using Vectashield with DAPI (Vector Laboratories, Burlingame, CA) and examined using a FluoView FV10i confocal microscope system (Olympus, Center Valley, PA) at 488 nm (green), 594 nm (red) and 405 nm (blue).

## Results

### Collagen genes *COL1A1*, *COL1A2*, and *COL3A1* are highly expressed in fibroblasts relative to OSCC cells

**Fig 1:**
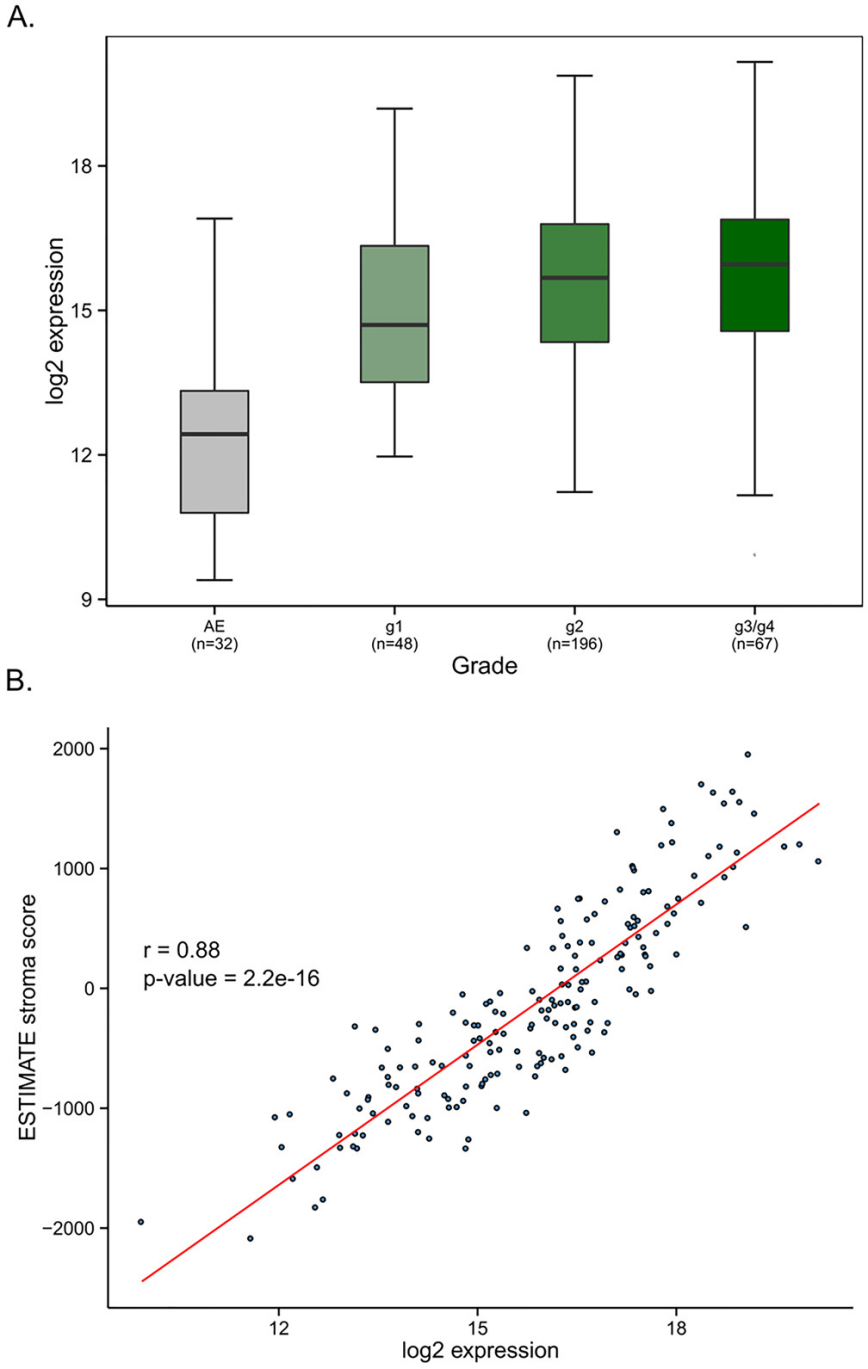
Collagen expression is elevated early in OSCC and can be used to identify potential OSCC CAF markers. **A**. Boxplot of average fibroblast-specific collagen expression (*COL1A1*, *COL1A2*, *COL3A1*) with respect to tumor grade for TCGA OSCC data. **B**. Correlation of stroma scores obtained using the ESTIMATE algorithm with mean collagen expression (Pearson r = 0.88, p-value < 0.0001). AE: Adjacent normal epithelium

Collagen type I, which is typically produced by fibroblasts, is the most abundant protein in human body and the main component of various connective tissues, including tumor stroma [12–14]. CAFs are the principal producers of collagen and other ECM proteins in tumor stroma [12,13]. Because type I collagen fibrils are usually associated with collagen type III we selected *COL1A1*, *COL1A2*, and *COL3A1 genes a*s representative markers of CAFs. We first validated expression of *COL1A1*, *COL1A2*, and *COL3A1*, in OSCC fibroblast pairs isolated from tumor or its adjacent stroma, as well as in two OSCC tumor cell lines, CAL27 and HSC-3, using quantitative PCR. We verified that the collagen genes were expressed at high levels specifically in the fibroblasts (S1 Table). Of note, the mean collagen expression in the TCGA OSCC cohort was higher in tumor samples compared to adjacent, morphologically normal epithelia (AE), with a sustained elevated expression in tumor grade 1 to grade 3/grade 4 groups, suggesting an early activation of fibroblasts in primary OSCCs (ANOVA p-value < 0.0001. Fig 1A).

### Mean collagen expression strongly correlates with ESTIMATE stromal scores in OSCC

To independently assess the reliability of collagen expression as a proxy for stromal infiltration, we correlated stromal score values generated using the ESTIMATE algorithm with the corresponding mean collagen expression. Remarkably, we found that the mean expression of the three chosen collagen genes was highly positively correlated (Fig 1B; Pearson r = 0.88, p-value < 0.0001) with the ESTIMATE-based stromal scores derived using a gene signature comprising a total of 141 genes. Despite strict selection for inclusion, TCGA tissue samples may retain a heterogeneous mix of cell types, causing varying levels of tumor purity. To assess whether the expression of the three specific collagens simply reflected the level of overall tumor purity, we correlated the average collagen expression and OSCC tumor purity estimated using the ABSOLUTE algorithm. To this end, TCGA samples with matching RNASeq expression and ABSOLUTE-based tumor purity estimates (N = 278) were analyzed. We observed a moderate negative correlation between the two features (r = -0.32; p-value = 4.4e-08. S1 Fig), indicating that higher average fibroblast-specific collagen expression only partially explains corresponding lower tumor purity. Degree of immune cell infiltration would be a logical additional contributor to reduced tumor purity not captured by collagen expression. Taken together, these results suggest that, in the context of OSCC, the mean expression of these three collagens may serve as a good proxy for stromal infiltration.

### Identification of potential stromal markers in OSCC

**Fig 2:**
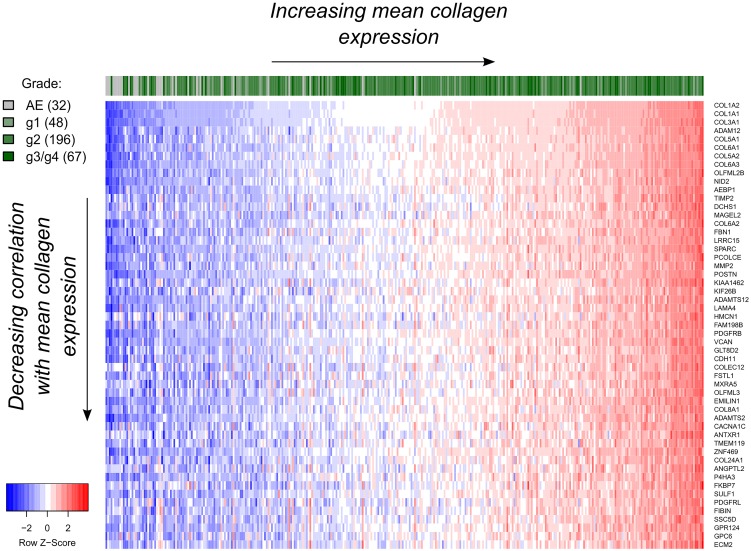
Gene expression profile of the top 50 hits (53 including *COL1A1*, *COL1A2* and *COL3A1*) obtained from the nearest neighbor (NN) gene expression analysis using mean collagen expression as a reference for TCGA OSCC data. Only samples with known tumor grade are highlighted. Expression values were log_2_-transformed after adding a pseudo-count of 1 and standardized per gene prior to their visualization. AE: Adjacent normal epithelium.

We performed a genome-wide 'nearest neighbor' (NN) analysis using the TCGA OSCC dataset to identify genes whose expression best-correlated with the average expression of the three chosen collagen genes (see Genome-wide collagen Nearest Neighbor (NN) analysis and S2 Table). Among the top-ranking genes, we found primarily ECM-specific genes, such as collagen type VI (*COL6A1*, *COL6A3*), *FBN1*, *ADAM12*, *SPARC*, and *POSTN* (Fig 2). *PDGFRB* was also found to be highly positively correlated (Pearson r = 0.85, FDR = 0.0085), and was the highest-ranked transmembrane protein-encoding gene among the 20,243 genes tested (rank = 25), suggesting that PDGFRβ could serve as a cellular marker of OSCC CAFs. Gene-set enrichment analysis (GSEA) confirmed a positive enrichment of genes involved in ECM organization and signaling by PDGF (S3 Table) with respect to the average collagen expression. Also, the top 50 genes were found to be more highly correlated with each other within the OSCC sample group than within the AE samples (S2 Fig), suggesting a more coordinated expression of these genes in OSCCs compared to controls. Expression of select genes within the top 50 hits was further examined experimentally using quantitative PCR in OSCC fibroblast pairs and in OSCC tumor cell lines (S1 Table). Whereas all of the assessed genes showed high expression in fibroblasts, several genes, including *SPARC*, *ADAM12*, *TIMP2*, *MMP2*, and *COL5A2* were also expressed in carcinoma cells, albeit at lower levels. The remaining genes were either undetected or expressed at negligible levels in tumor cells, confirming their CAF-specific expression. It is important to note that gene expression in tumor cell lines may not accurately reflect *in vivo* conditions, and that this should be further experimentally validated in patient tumors.

### PDGFRβ is a reliable marker of CAFs in OSCC

**Fig 3:**
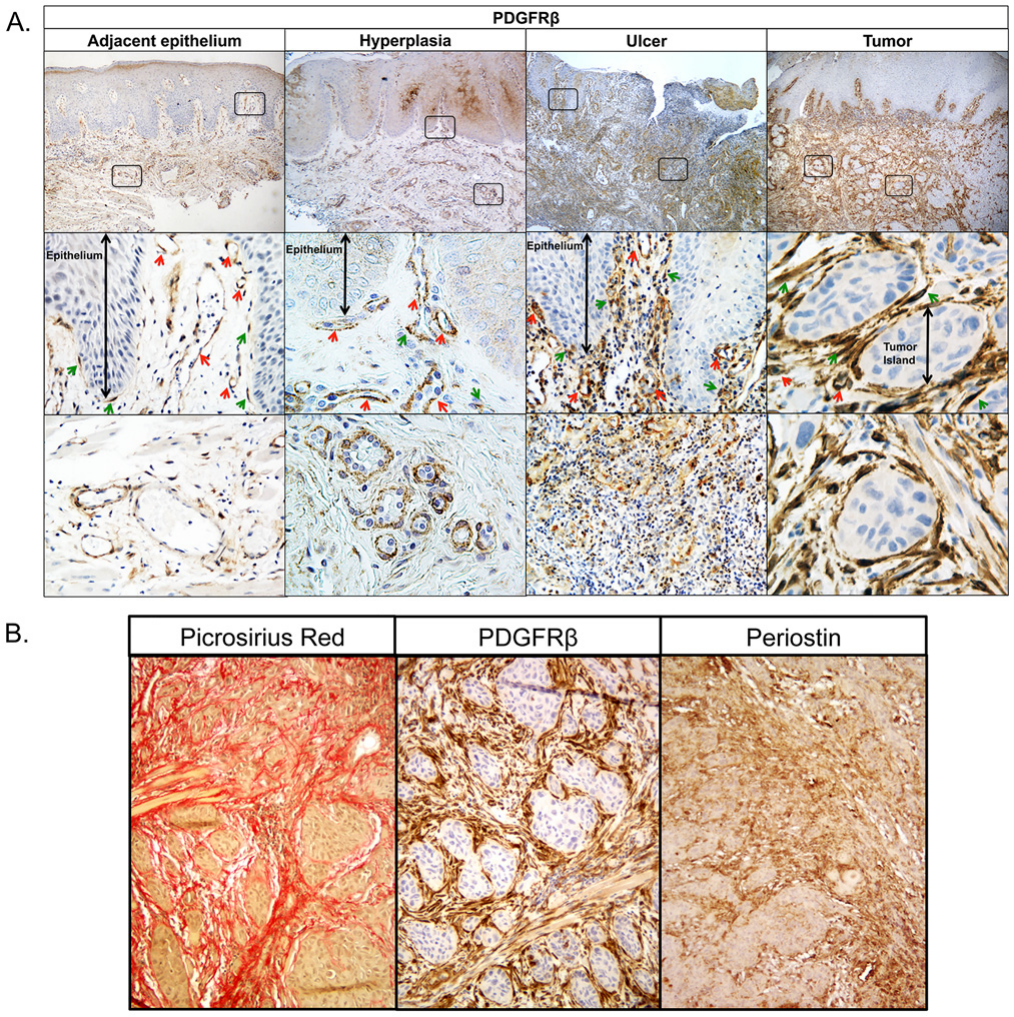
PDGFRβ localizes primarily to the surrounding stroma in OSCC. **A**. PDGFRβ staining is absent in adjacent epithelium and hyperplasia samples, but is present in ulcer and OSCC specimens. Red arrows indicate blood vessels and green arrows indicate fibroblasts. **B**. Picrosirius red staining of collagen 1 and 3 fibers (left) and immunostaining of PDGFRβ (middle) and additional marker periostin (right) that were identified from the NN analysis.

To assess the expression and tissue localization of PDGFRβ in OSCC, we performed immunostaining in twelve OSCC tumor tongue specimens and paired morphologically normal AE tissue. For comparison, we analyzed PDGFRβ expression in six tongue specimens representing benign hyperkeratosis and epithelial hyperplasia, as well as 10 tongue ulcer specimens. PDGFRβ localized to the blood vessels in AE tissue, consistent with a pericyte/smooth muscle cell expression (Fig 3A). Similar expression patterns were also observed in the hyperkeratosis/hyperplasia specimens. Conversely, numerous fibroblasts expressing high levels of PDGFRβ were found in stroma surrounding tumor islands in all OSCC specimens (12/12), while no PDGFRβ expression was detected on tumor cells. Note also the very close proximity of PDGFRβ-positive cells to carcinoma cells. An increased presence of the PDGFRβ-positive cells was also observed in ulcer (10/10) in the granulation tissue, which forms during the healing process. PDGFRβ was primarily expressed on fibroblasts, as well as cells surrounding capillaries and small blood vessels, and, occasionally, on dendritic cells. These observations are consistent with the notion that there are many similarities between inflammatory wounds such as ulcers and activated tumor stroma [5].

Since periostin (POSTN) was among the ECM genes that were closely associated with the collagens (rank = 18, Pearson r = 0.88) and PDGFRβ in the TCGA analysis, we also examined its expression in OSCC specimens. POSTN was abundant in collagen-rich stromal regions identified using picrosirius red staining and its expression overlapped with that of PDGFRβ (Fig 3B).

**Fig 4:**
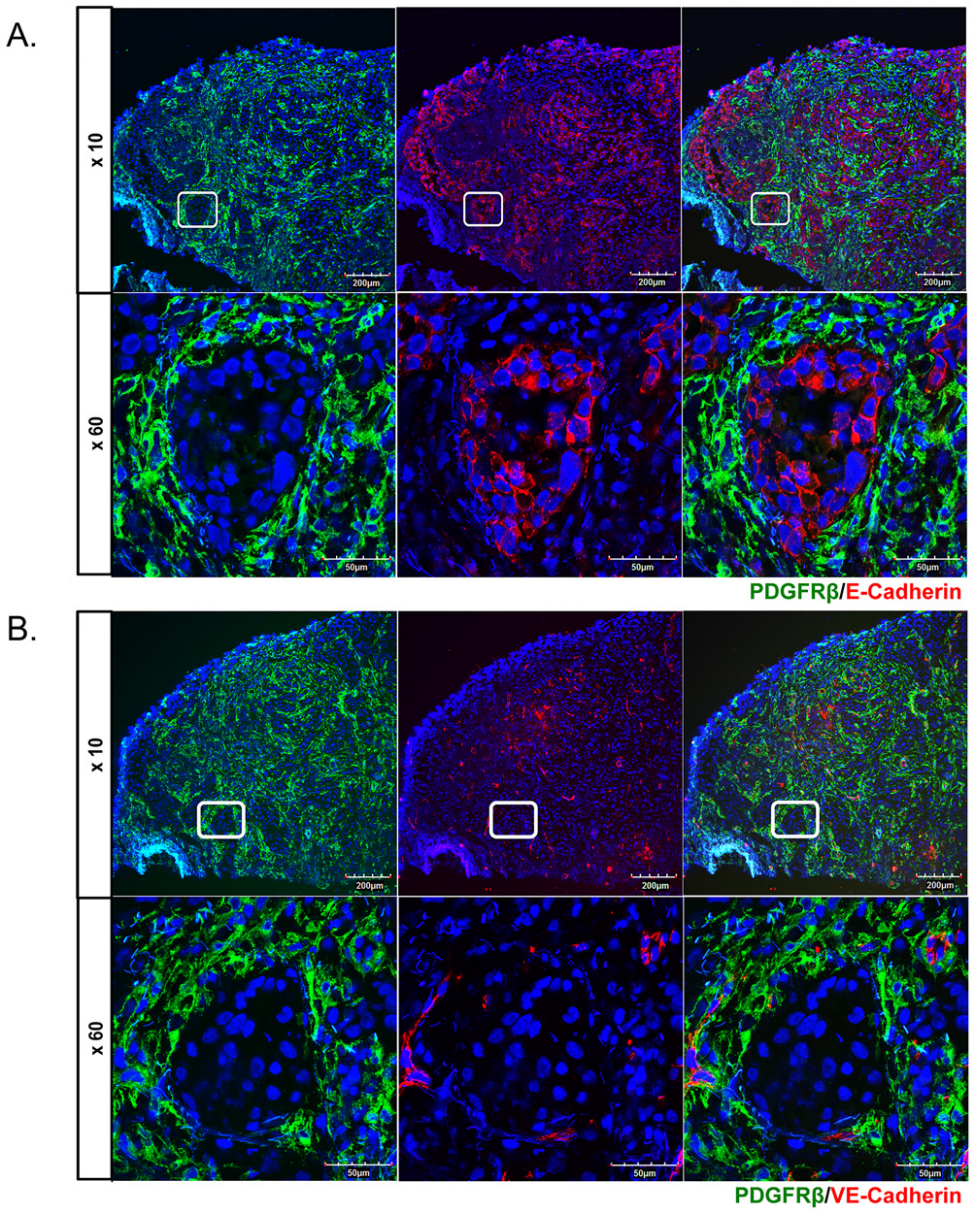
PDGFRβ does not co-localize with tumor cells and endothelium. Double immunofluorescence staining of PDGFRβ/E-Cadherin (**A**.), and PDGFRβ/VE-Cadherin (**B**.) on cryosections from oral tumor biopsies. Representative images are shown for four tumors.

Given the close proximity of PDGFRβ-positive cells to carcinoma cells we wished to determine whether carcinoma cells in the invasive tumor fronts also express PDGFRβ. Tumor specimens were double stained for PDGFRβ and a representative marker of carcinoma cells, E-cadherin. As shown in Fig 4A there was no co-localization between these two markers. Because PDGFRβ can be expressed in pericytes, as well as fibroblasts we next examined co-localization of PDGFRβ with VE-cadherin, a marker of endothelial cells. VE-cadherin positive cells were primarily found in the stromal compartments in close proximity to PDGFRβ positive cells, which may represent pericytes (Fig 4B). There was no co-localization between these two markers.

As αSMA [7] and podoplanin [15] were previously reported to be expressed in OSCC CAFs, these markers were considered in our analysis. Of note, neither αSMA nor podoplanin showed as high a correlation with the average collagen gene expression in the TCGA analysis (Pearson r = 0.51 and 0.21 respectively). Expression of αSMA in unaffected tongue tissues, hyperplasia and ulcer specimens was localized to pericytes/SMA cells around the blood vessels (S3 Fig). Furthermore, in contrast to the prevalent PDGFRβ expression, αSMA-positive CAFs were only found in a subset of OSCC specimens (3/11) (S3 Fig). It has been reported that αSMA is usually associated with moderately and poorly differentiated OSCC, but is infrequent in well differentiated tumors[16] and our results are consistent with this finding. Podoplanin was expressed primarily in lymphatic endothelial cells (S4 Fig). In OSCC, podoplanin was weakly expressed in a subset of CAFs (3/11) and overlapped with that of αSMA (S4 Fig). Since PDGFRβ and αSMA are also expressed in pericytes their increased expression during tumor progression may, in part, reflect tumor-induced angiogenesis.

### PDGFRβ is a potential CAF marker in multiple cancers

**Fig 5:**
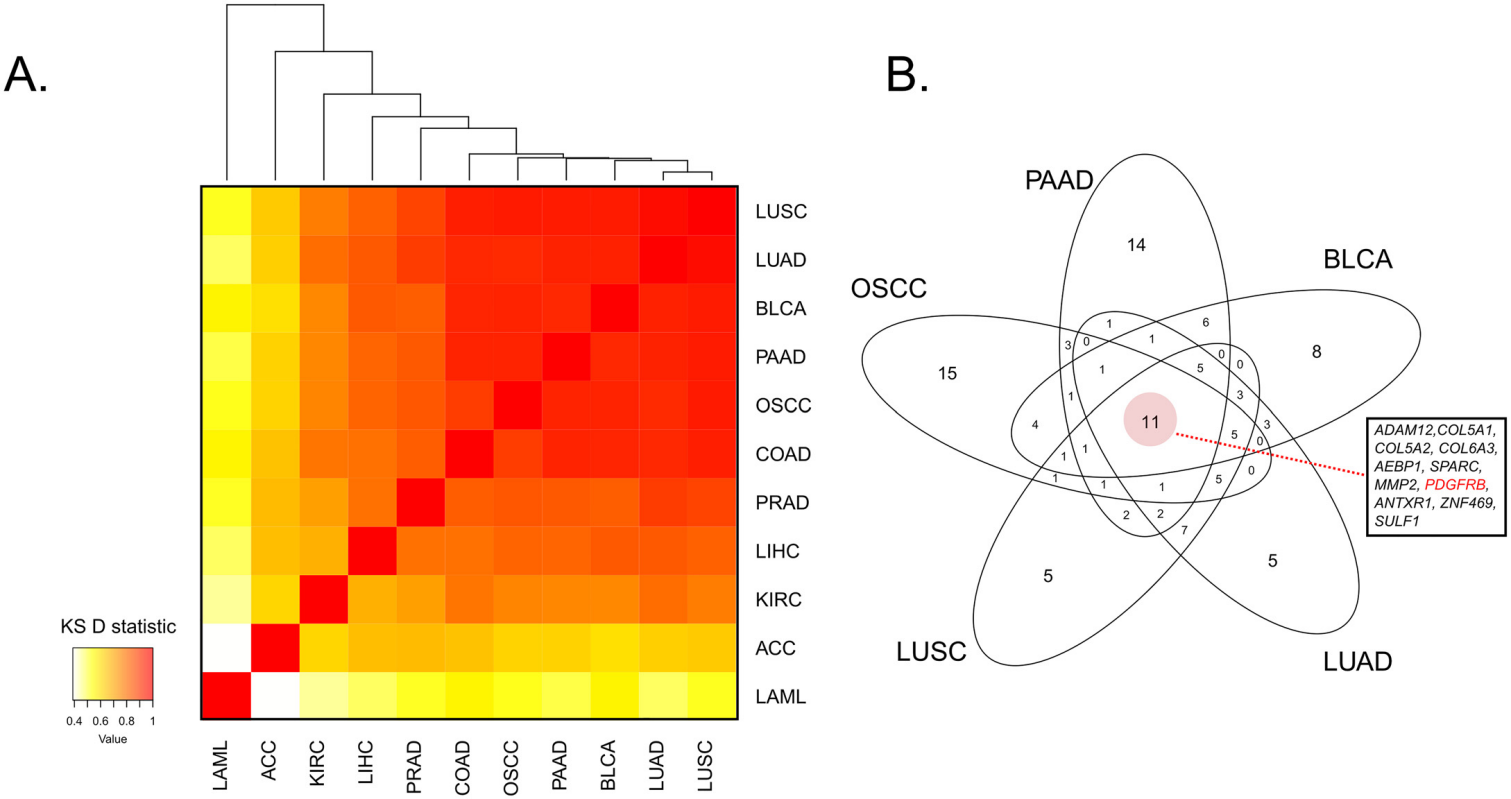
Pan-cancer NN analysis highlights PDGFRβ as a potential CAF marker in multiple cancer types A. Heatmap of average pairwise Kolmogorov-Smirnoff (KS) D statistics using the top 50 genes in the collagen NN cancer analysis of each of 11 cancer types. **B**. Venn diagram of top 50 genes in collagen NN ranked list within the five most closely related cancers (based on the average KS D statistic; Fig 5A). Core overlap has 11 genes, which includes PDGFRβ, highlighted in red. ACC: Adrenocortical carcinoma; BLCA: Bladder urothelial carcinoma; COAD: Colon adenocarcinoma; KIRC: Kidney renal clear cell carcinoma; LAML: Acute myeloid leukemia; LIHC: Liver hepatocellular carcinoma; LUAD: Lung adenocarcinoma; LUSC: Lung squamous cell carcinoma; PAAD: Pancreatic adenocarcinoma; PRAD: Prostate adenocarcinoma.

From our NN analysis within OSCC TCGA data, we considered other potential CAF markers, including PDGFRα, FSP1/S100A4, VIM, and FAP; however, none of these markers showed as close an association with the average collagen expression as PDGFRβ (S2 Table). To assess how closely related different cancer types are with respect to their collagen-associated expression signatures, we extended the described NN analysis to 10 additional TCGA cancer datasets (see Pan-cancer collagen NN enrichment analysis). These included nine carcinoma datasets, as well as a leukemia dataset (LAML) as a negative control of ECM behavior owing to its non-solid tissue origin. Clustering based on the similarity of their NN rankings with respect to average collagen expression as measured by a pairwise KS enrichment statistic yielded a clear segregation of cancer types, with most manifesting a high similarity in their list of collagen-correlated markers, and with LAML the only clear outlier, likely reflecting its non-solid origin (Fig 5A). Of note, the overlap between the top 50 genes of the five most closely related cancer types (OSCC, LUAD, LUSC, BLCA and PAAD, Fig 5A), yielded a set of 11 genes that included *PDGFRB* (Fig 5B). Furthermore, *PDGFRB* was consistently found to be within the top 50 hits of each NN ranked list for a total of eight out of the 11 cancer types (with the exceptions being KIRC, LAML and LIHC), with a mean genome-wide rank of 28.5. Its correlation with mean collagen expression was statistically significant (FDR ≤ 0.05) in all tumor types but LAML (FDR = 0.089).

## Discussion

It is becoming increasingly accepted that CAFs and their products may promote several hallmarks of cancer, including those contributing to tumor invasion and metastasis [17]. The presence of CAFs could thus serve as a potential prognostic marker in multiple cancers, including OSCC. Identification of CAFs remains challenging due to the heterogeneity of CAF markers across different cancer types. Our novel approach, combining a high-throughput gene expression analysis of large primary tissue datasets with experimental validation of OSCC specimens, permits relatively rapid and comprehensive characterization of CAF markers without having to resort to large-scale immunostaining experiments. Taken together, our results suggest that PDGFRβ is the most consistent cellular marker of stromal activation both during early and late stages of OSCC progression, and could be utilized for the isolation and further characterization of stromal fibroblasts in OSCC. Our analyses also suggest that PDGFRβ may be similarly relevant in additional carcinomas, however, this should be further investigated.

A large study of several tumors, which did not include OSCC, systematically evaluated expression of PDGFRβ as well as PDGFRα on fibroblasts and pericytes by immunohistochemistry [18]. The data revealed a variable expression of these receptors in different tumors, with fibroblast expression of PDGFRβ most common in lung and colon tumors. Interestingly, stromal expression of PDGFRβ in breast cancer correlated with poor prognosis [18]. It is important to note that in our study PDGFRβ was not expressed on carcinoma cells, however with the limited number of specimens analyzed, we cannot exclude a possibility that PDGFRβ may be expressed in a subset of OSCCs, as has been shown for other cancers [19,20]. Relevant to our findings, previous studies demonstrated elevated expression of PDGF primarily in tumor cells in OSCC [21,22], suggesting a paracrine mechanism of fibroblast recruitment through a PDGF-PDGFRβ axis. Interestingly, many of the top-ranking ECM-specific genes in our analysis have previously been evaluated in OSCC and linked to tumor progression. Of particular interest are matricellular proteins, SPARC and POSTN. Elevated expression of both genes was documented in OSCC [23] and correlated with the presence of metastases [24,25]. Furthermore, basal lamina components, NID2 and LAMA4 were previously investigated in OSCC [26,27]. Franz et al. reported that stromal expression of laminin α4 chain, which is undetectable in healthy and hyperplastic mucosa, gradually increased in tumor grades 1 to 3 [26]. On the other hand, increased hypermethylation of NID2 promoter was observed in OSCC samples, indicative of decreased gene expression [27]. However, expression of NID2 in the TCGA OSCC dataset was higher in tumor samples compared to adjacent normal epithelia with a sustained elevated expression in tumor grades 1 to 3 (data not shown). It would be important to experimentally evaluate expression of NID2 mRNA and protein in OSCC specimens to clarify this inconsistency.

Matrix metalloproteinases are multifunctional proteins that are central to tumor progression and metastasis [28]. MMP2 was the highest-ranking MMP gene whose expression was found to significantly correlate with the collagen genes in our NN analysis. Elevated expression of MMP2 was previously reported in OSCC and was associated with more invasive phenotype [29]. Another proteolytic family of enzymes related to MMPs is a disintegrin and metalloproteinase (ADAM) multifunctional gene family, which also regulates cancer cell proliferation and progression [30]. In particular, ADAM12 is overexpressed in various pathological conditions, including adenocarcinoma and breast and bladder cancer, as well as several fibrotic diseases [31]. In OSCC, increased expression of ADAM12 mRNA and protein has been observed and correlated with primary tumor size and tumor stage [32]. Two other related family members ADAMTS12 and ADAMTS2 were also among the top ranking genes in our NN analysis. Interestingly, ADAMTS12 has been shown to have both pro- and anti-tumorigenic activities, while very little is still known about the role of ADAMTS2 in cancer [33]. To date, there are no reports on the expression of ADAMTS family members in OSCC. In addition to the genes discussed, there were additional stromal genes identified by the NN analysis, including *CDH11*, *OLML3*, *FSTL1*, *AEBP1*, *VCAN*, previously reported to correlate with tumor progression and metastasis in various cancers [34–36]. Elevated expression of CDH11 mRNA has indeed been observed in OSCC [23].

The altered expression of stromal markers is consistently associated with poor prognosis. However, despite the increased interest in this field, the specific functional role of the stromal alterations in facilitating tumor progression is still poorly understood. A major limitation in studying CAF populations *in vivo* has been the lack of robust fibroblast markers that are both fibroblast-specific, and expressed in all fibroblast populations [12]. Our study provides a novel, unbiased approach to select CAF markers with relevance to OSCC for further validation and mechanistic studies, with the ultimate goal of identifying novel therapeutic targets for OSCC.

## Supporting Information

S1 Fig Scatter plot of mean collagen expression *vs*. tumor purity estimates derived using the ABSOLUTE algorithm highlighting a moderate negative correlation (Pearson r = -0.32, p-value < 0.0001). (TIFF)

S2 Fig Pairwise gene expression Pearson correlation of the top 50 hits from collagen NN analysis within (A.) tumor and (B.) control TCGA OSCC samples. AE: Adjacent normal epithelium.
(TIFF)

S3 Fig Immunostaining highlighting localization of alpha smooth muscle actin (αSMA) in adjacent normal oral epithelium (AE), hyperplasia, ulcer, and OSCC tongue specimens. (TIFF)

S4 Fig Immunostaining highlighting localization of podoplanin in adjacent normal oral epithelium (AE), hyperplasia, ulcer, and OSCC tongue specimens. (TIFF)

S1 Table Quantitative PCR expression for genes among top hits of collagen-based nearest neighbor analysis using fibroblasts isolated from OSCC specimens (RF), their adjacent epithelium (AE), CAL27 and HSC-3 cells. Fibroblast to cancer expression for each gene was calculated by averaging RF and AE, and CAL27 and HSC-3 values, respectively, normalizing the former value to 1. Genes are sorted in decreasing order of Fibroblast to cancer expression, with GAPDH used as an internal control. NA: Undetected.
(XLSX)

S2 Table Collagen-based nearest neighbor analysis results in OSCC. Genes are ranked in decreasing order of their Pearson correlation with respect to the mean expression of *COL1A1*, *COL1A2* and *COL3A1*. FDRs indicate permutation-based significance estimates after multiple testing correction. The Gene_ID field represents both official HGNC gene symbols and Entrez gene IDs for each gene.
(XLSX)

S3 Table Gene-set enrichment analysis (GSEA) result table highlighting gene-sets positively enriched with respect to average collagen expression in OSCC. TCGA OSCC data was queried against canonical pathway genesets (c2.cp.v5.0) derived from the molecular signature database (MSigDb) using average collagen expression as a continuous ranking variable.
(XLSX)
