# Supplementary figures and images for: PDGFRβ Is a Novel Marker of Stromal Activation in Oral Squamous Cell Carcinomas
Source: PLoS One. 2016 Apr 29;11(4):e0154645. doi: 10.1371/journal.pone.0154645 (PMC4851360; doi:10.1371/journal.pone.0154645)

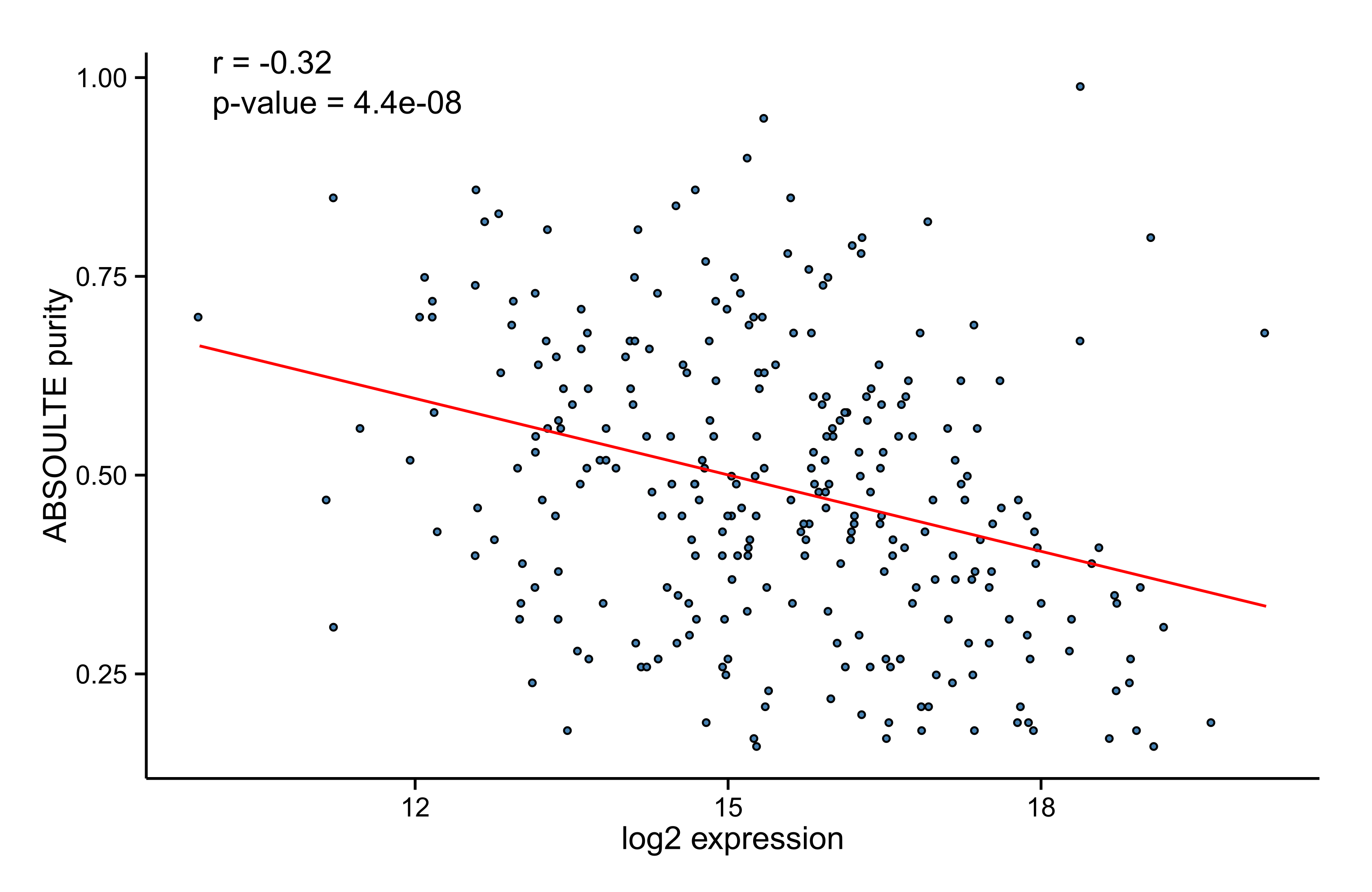

Supplement: S1 Fig — (TIFF) [file pone.0154645.s001.tiff]

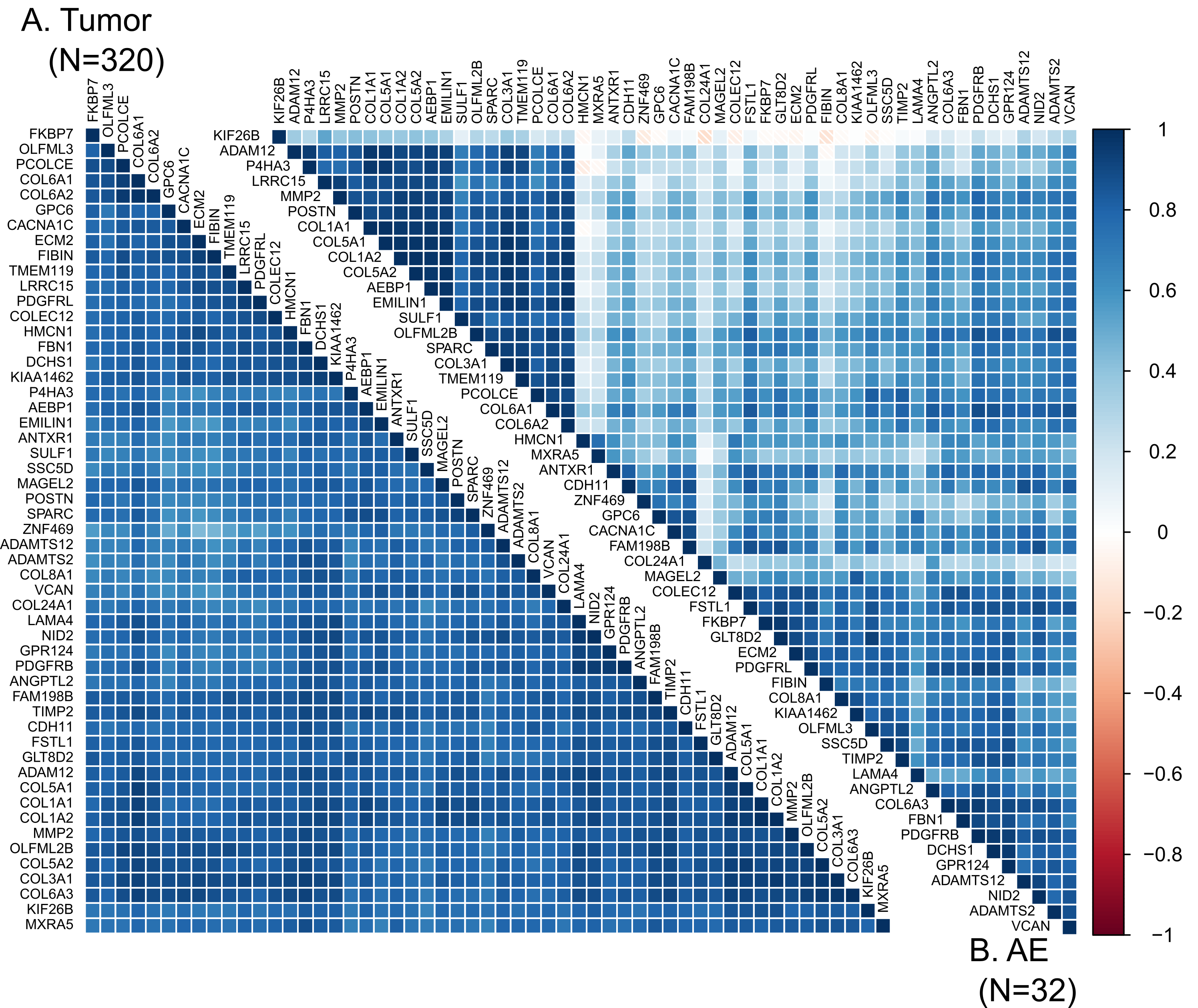

Supplement: S2 Fig — AE: Adjacent normal epithelium. (TIFF) [file pone.0154645.s002.tiff]

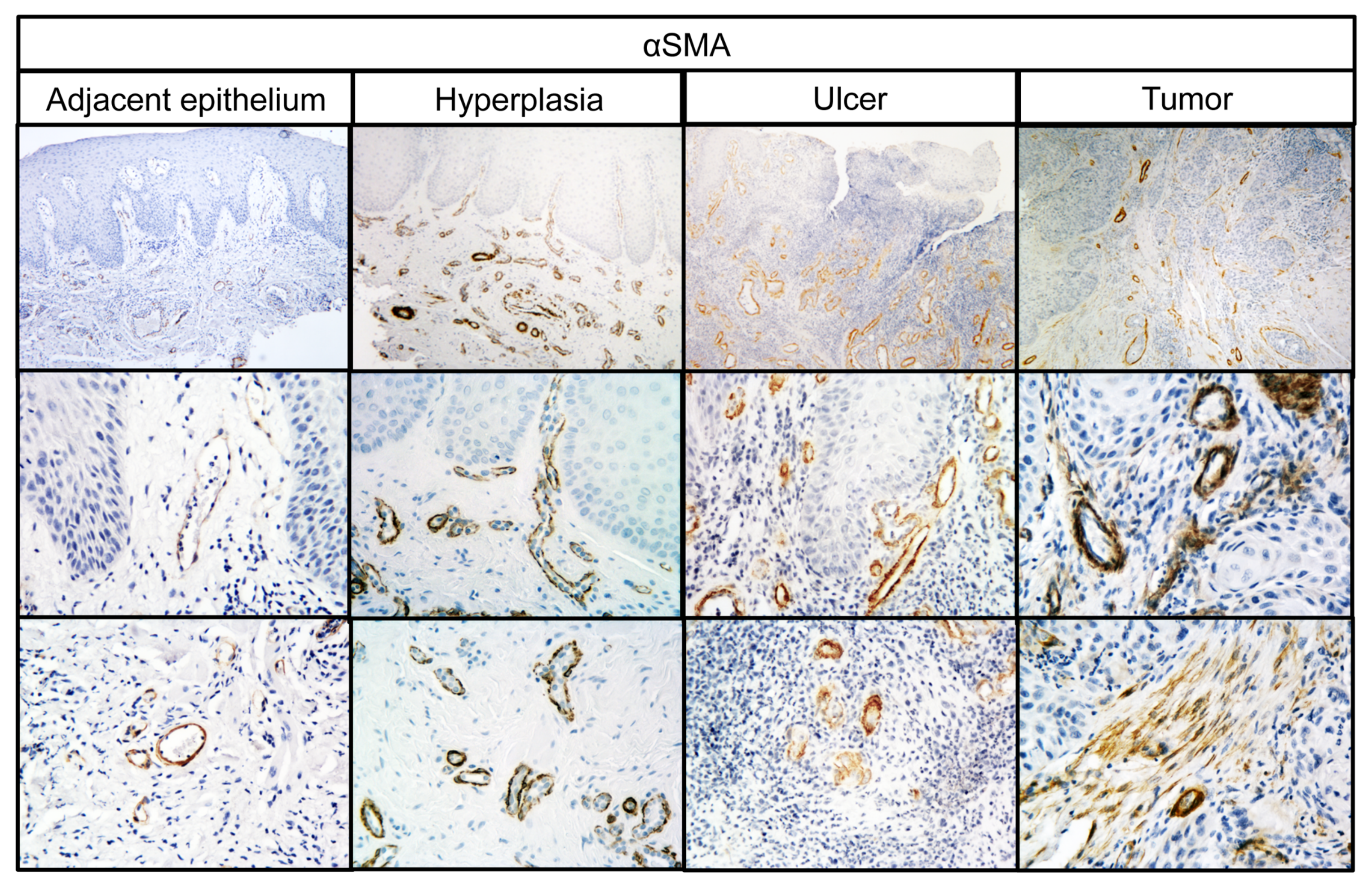

Supplement: S3 Fig — (TIFF) [file pone.0154645.s003.tiff]

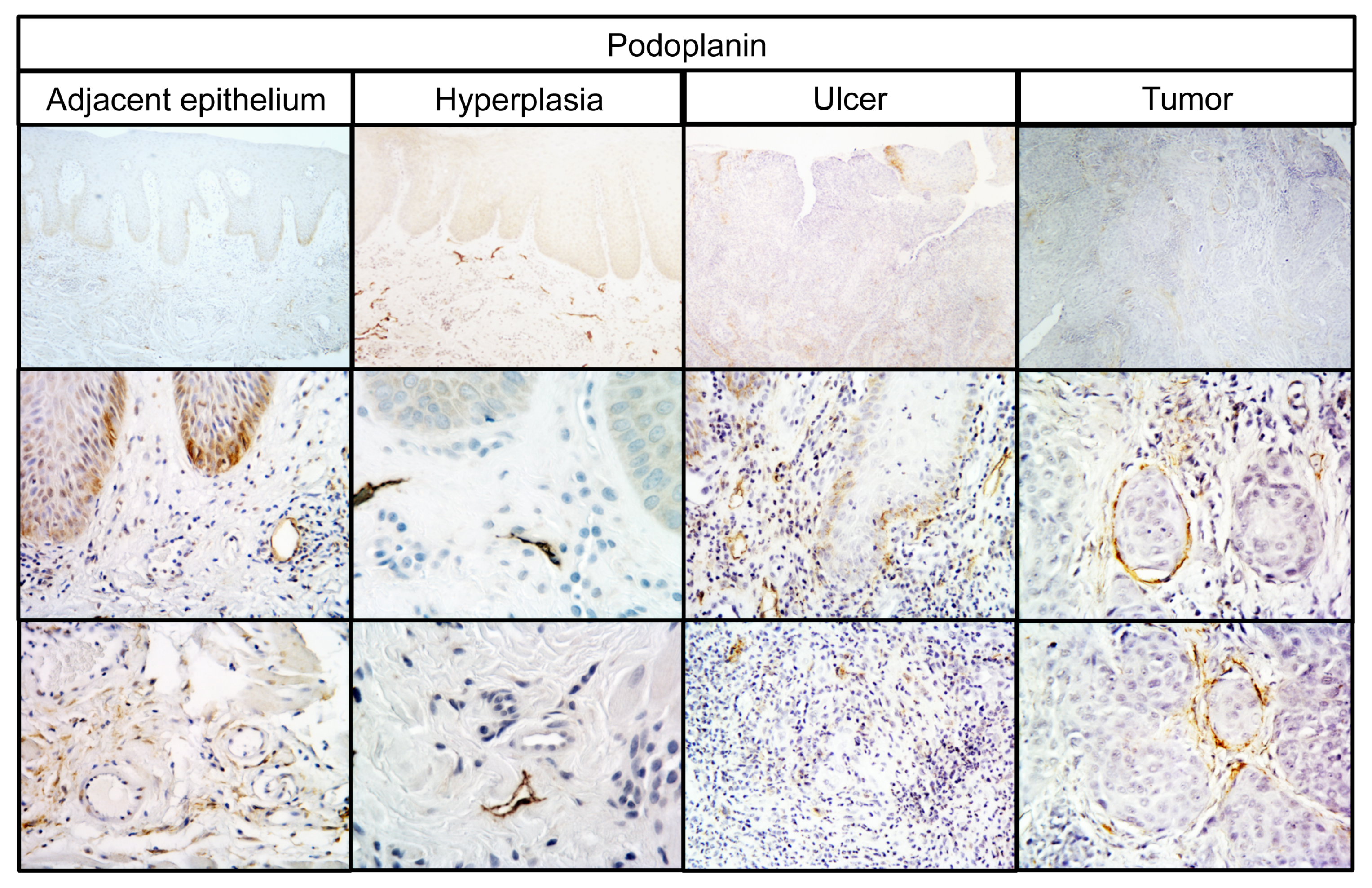

Supplement: S4 Fig — (TIFF) [file pone.0154645.s004.tiff]
